# ElectroMotive drug administration (EMDA) of Mitomycin C as first-line salvage therapy in high risk “BCG failure” non muscle invasive bladder cancer: 3 years follow-up outcomes

**DOI:** 10.1186/s12885-018-5134-7

**Published:** 2018-12-06

**Authors:** Marco Racioppi, Luca Di Gianfrancesco, Mauro Ragonese, Giuseppe Palermo, Emilio Sacco, Pier Francesco Bassi

**Affiliations:** 1 Department of Urology, Fondazione Policlinico Universitario “A. Gemelli” IRCSS, Largo Agostino Gemelli, 8, 00168 Rome, Italy; 2 Università Cattolica del Sacro Cuore, L.go A. Gemelli, 8, 00168 Rome, Italy

**Keywords:** EMDA, Device-assisted therapy, High risk non muscle-invasive bladder cancer, BCG failure, Mitomycin C, Salvage therapy, Sparing bladder

## Abstract

**Background:**

In case of high grade non-muscle invasive bladder cancer (HG-NMIBC), intravesical BCG represents the first-line treatment; despite the “gold” standard therapy, up to 50% of patients relapse, needing radical cystectomy. Hence, alternative therapeutic strategies have been developed. The aim of the study was to evaluate a first-line salvage treatment with EMDA®-MMC in patients with HGNMIBC unresponsive to BCG.

**Methods:**

We carried out a prospective, single-center, single-arm Phase II study in order to evaluate the efficacy (in terms of recurrence and progression) and the safety of the EMDA®-MMC treatment in 26 (21 male, 5 female) consecutive patients with “BCG refractory” HGNMIBC on a 3 years follow-up.

EMDA®-MMC treatment consisted of 40 mg of MMC diluted in 100 ml of sterile water retained in the bladder for 30 min with 20 mA pulsed electric current.

EMDA®-MMC regimen consisted of an induction course of 6 weekly instillations followed by a maintenance course of 6 monthly instillations.

Follow-up was performed with systematic mapping biopsies of the bladder (with sampling in the prostatic urethra for men), voiding and washing urinary cytology, radiological study of the upper urinary tract.

We performed Survival Kaplan-Meier curves and Log-rank test in order to analyze high grade disease-free survival.

**Results:**

At the end of follow-up, 16 patients (61.5%) preserved their native bladder; 10 patients (38.4%) underwent radical cystectomy, in 6 patients (23.1%) for recurrent HGNMIBC and in 4 patients (15.4%) for progression to muscle-invasive disease.

At the end of follow-up, stratifying patients based on TNM classification (TaG3, T1G3, Cis, TaT1G3 + Cis), disease-free rates were 75, 71.4, 50 and 25%, respectively; survival curves showed statistically significant differences (*p* value < 0.05).

Regarding toxicity, we reported severe adverse systemic event of hypersensitivity to the MMC in 3 patients (11.5%), and local side effects in 6 patients (26.1%).

**Conclusions:**

In the field of alternative strategies to radical cystectomy, the EMDA®-MMC could be considered safe and effective in high-risk NMIBC unresponsive to BCG, as a “bladder sparing” therapy in selected patients. Multicenter studies with a larger number of patients and a longer follow-up might confirm our preliminary results.

**Trial registration:**

EudraCT2017-002585-43. 17 June 2017 (retrospectively registered).

## Background

Intravesical Bacillus of Calmette Guerin (BCG) represents the first-line treatment in case of high grade non muscle invasive bladder cancer (HG-NMIBC), following trans-urethral resection of bladder tumor (TURBT) [1, 2]. Despite the “gold” standard therapy, about 40-50% of these patients relapse. The BCG failure could represent a challenge in the common clinical practice, which implies difficult choices of the best-tailored treatment.

Intravesical immunotherapy with BCG induces a variety of immune responses that correlate with anti-tumor activity and it is known that in case of intermediate-high risk NMIBC BCG is more effective than intravesical chemotherapy in prevention of recurrence and progression, although it is more toxic [3–7].

The indication to radical cystectomy becomes imperative in case of persistent disease after an intravesical immunotherapy course. However, in clinical practice surgical therapeutic choice could not be so obvious due to the will of the patient to preserve the bladder or due to the unfitness for surgery of patients with multiple comorbidities. With the aim to preserve the bladder (new) alternative therapeutic strategies to early radical cystectomy have been developed, always taking into account the aggressiveness of HG-NMIBC [8–15].

EMDA®-MMC (ElectroMotive Drug administration of Mitomycin C) is one of these choices. This device-assisted therapy applies an electrical current to induce a directional and accelerated movement of the ionized drug toward the tissue, expressing a greater local therapeutic effect and increasing its effectiveness [16, 17]. EMDA®-MMC is characterized by a combination of different electromolecular interactions that improves drug absorption: iontophoresis, electroosmosis/electrophoresis and electroporation might increase effectiveness for an improved bladder wall penetration of MMC compared to passive way [17].

A special catheter with a spiral electrode (anode) is inserted into the bladder and two electrodes (cathode) are placed on the suprapubic region. The current generator is connected to the electrodes and polarity, amperage and duration of treatment are set.

Several in vitro and in vivo studies demonstrated how MMC concentrations are significantly increased in the bladder wall by applying an electric current. Di Stasi et al. reported a drug concentration 4 to 7 times greater of electromotive MMC compared to passive MMC, explaining the improved response rates [18, 19].

Brausi et al. evaluated EMDA®-MMC efficacy and toxicity in multifocal G1-G2 Ta-T1 urothelial bladder cancer patients: at 8 weeks, rates of complete response were no different compared to passive MMC but there were lower recurrence rates and longer disease-free intervals, with low additional side effects, with EMDA®-MMC treatment [20].

Moreover, Di Stasi et al. reported EMDA®-MMC efficacy and toxicity compared to passive MMC and BCG; at 3 and 6 months, they reported higher EMDA®-MMC response rates compared to passive MMC (at the cost of more side effects) and non-inferior compared to BCG (also with fewer side effects) [21].

On this background, the aim of this study was to explore the role of EMDA®-MMC as first line salvage treatment in selected patients with HGNMIBC unresponsive to intravesical BCG, with a follow-up of 3 years [22].

## Methods

A prospective, non-controlled, non-randomized, single arm, single center phase II clinical trial was carried out in order to estimate the efficacy and toxicity of a (new) treatment.

Phase II clinical trials are only able to detect a large treatment improvement due to their small sample size; indeed, it is not always possible to recruit large sample size for the reduced number of patients eligible for this kind of studies.

In this study we tried to achieve the main aims of Phase II clinical trial: to evaluate the pros and the cons of a therapeutic intervention for a subgroup of patients with a specific type of cancer.

Based on the improved response of this treatment compared to the standard intervention obtained with this type of trial, further investigations with large scale randomized phase III clinical trial might be warranted.

Due to exploratory nature of the study, we did not perform a formal sample size calculation and we mainly included “clinical practice” considerations for this issue: efficacy and safety as well as proof-of-concept (PoC) regarding intravesical EMDA®-MMC treatment.

The number of 26 consecutive patients with BCG-refractory HGNMIBC referred to our institution was assumed to be adequate to gain information on efficacy (primary outcome, in terms of recurrence rate and progression rate) and safety (secondary outcome) of intravesical EMDA®-MMC treatment.

BCG-refractory HGNMIBC is mainly and widely treated with RC; the trial evaluated a few number of patients refusing major surgery even if they were appropriately informed in a shared decision-making process about the international guidelines recommendation on oncological significance of early RC; to date, treatments other than RC should be considered oncologically inferior in BCG failure patients [1, 23–27].

Due to the complex administration of the intravesical EMDA®-MMC this trial was performed in a single center with a solid experience in clinical research as well as in clinical management of patients with bladder cancer, with particular regard in intravesical conservative alternative approaches to RC.

All patients had BCG-refractory HG-NMIBC. We considered EAU (European Association of Urology) [1], FICBT (First International Consultation on Bladder Tumors) [26] and IBCG (International Bladder Cancer Group) [27] guidelines and we assumed BCG-refractory HG-NMIBC definition according to EAU guidelines.

From October 2012 to March 2016, we analyzed 26 consecutive patients (21 male, 5 female) with an average age of 66 years (range 49-79) (Table 1).

**Table 1:** Patients characteristic

N° of patients	26
Age, mean years (range)	66 (49-79)
Gender, n° of patients (%)	
Male	21 (80.8%)
Female	5 (19.2%)
Months of follow-up, median	36
Histopathological stage, n° of patients (%)	
TaG3	4 (15.4%)
T1G3	14 (53.8%)
Cis	4 (15.4%)
Ta/T1G3 + Cis	4 (15.4%)
Time from first diagnosis, months range	3-9

All patients had an initial first diagnosis of HGNMIBC, therefore according to EAU guidelines all of them underwent at least an induction course (6 weekly intravesical doses) of BCG (EAU LE: 1a) due to its higher oncological efficacy than TURBT alone or TURBT+intravesical chemotherapy in this group of patients [28–32].

All patients had persistent high-grade NMIBC after BCG and were considered BCG-refractory; they were not disease-free within 6 months after BCG: 18 patients (4 patients with TaG3, 14 patients with T1G3) after a first weekly induction course and 8 patients (4 patients with Carcinoma in situ -Cis-, 4 with Ta/T1G3 + Cis) after a second weekly induction course at 3 months.

None of the patients underwent prior maintenance courses of BCG before the recruitment in the study. Furthermore, all patients with solitary or concurrent Cis underwent a subsequent additional induction BCG course after the previous one for evidence of persistent Cis at 3 months follow-up [33].

None of the patients underwent other intravesical chemotherapy or other experimental off-label treatment regimens besides BCG.

In this subgroup of patients, EAU guidelines recommendations consist of early radical cystectomy in order to obtain an optimal oncologic control [1].

In all patients, the BCG strain used was Seed RIVM by Medac® (derived from seed 1173-P2, 2 × 82 10 to 3 × 10 viable units). The Medac®-BCG powder was re-suspended with 50 ml of 0.9% normal saline and introduced into the bladder via a 10-12 French urethral catheter. Patients were instructed to hold the drug in the bladder for 2 h.

Four experienced urologists performed all the diagnostic cystoscopies, systematic mapping biopsies of the bladder and TURBTs. All specimens were analyzed by an experienced dedicated uropathologist. All Ta-T1 patients underwent a second resection (reTURBT), according to EAU guidelines.

In all patients, the histological specimen documented a pure urothelial cancer with detrusor muscle included in the resection; we did not analyzed patients with other histological variants.

The 2009 TNM classification [34] and the 2004 WHO grading system [35] were used for histologic reports.

Patients underwent an EMDA®-MMC induction course (40 mg of MMC diluted in 100 ml of sterile water retained in the bladder for 30 min with 20 mA pulsed electric current) of 6 weekly instillations.

Although several treatment times were studied in the literature [18–21, 36], we used the recommended treatment protocol consisting of a 30 min treatment duration.

At the end of the induction course, high-grade disease-free patients (16 patients, 61.5% of the total) underwent a maintenance course with monthly EMDA®-MMC for a total of 6 treatments.

Evaluation after treatment was done with systematic mapping biopsies of the bladder (with biopsies of the prostatic urethra for men), voiding and washing urinary cytology and radiological study of the upper urinary tract, forty-five days after the end of the induction/maintenance intravesical course and then every 3 months until the end of the follow-up.

Patients who did not benefit from conservative treatment with EMDA®-MMC underwent radical cystectomy.

A complete response (CR) was defined as the absence of any macroscopic neoplastic recurrence, a completely negative systematic mapping biopsies of the bladder with voiding and/or washing cytology negative for High Grade Urothelial Carcinoma (HGUC). A partial response (PR) was defined as the presence of low-grade papillary recurrence (down-grading) during follow-up; a partial response was assumed as a success of EMDA®-MMC in cancer treatment.

Both complete and partial responses were considered “high grade disease free” status in follow-up.

A neoplastic progression (NP) was defined as the presence of muscle-invasive bladder cancer (MIBC) during follow-up.

The secondary outcome was the assessment of toxicity; in case of grade 1 or 2 toxicity a symptomatic (empirical) therapy was administered until the remission of symptoms, while in case of cystitis or allergic reaction of grade 3 toxicity or dermatitis the immediate discontinuation of instillations with an endoscopic control were performed.

Inclusion criteria were: patients aged between 18 and 80 years, with evidence of recurrent high grade urothelial NMIBC, who just underwent at least one cycle of intravesical immunotherapy with BCG, with performance status 0-1 according to ECOG system, absence of urothelial tumor in upper urinary tract.

Exclusion criteria were: known allergy to the MMC, anatomic alterations of the lower urinary tract with significant urodynamic abnormalities, post-void residual volume greater than 100 ml, bladder capacity lower than 150 ml, urinary incontinence, previous pelvic radiotherapy, previous intravesical and/or systemic chemotherapy, known immune deficiency (HIV positive serology, patients in steroid or immunosuppressive therapy), active or uncontrolled urinary infections, liver, kidney or hematological function disorders, coexistence of any other primary tumor, patients unwilling or unable to collaborate for any reason with potential negative influence on follow-up, and pregnant or lactating women.

In order to analyze “high grade” disease-free survival Kaplan-Meier curves were built and a Log rank test was applied. The significant level was set at *p* value< 0.05.

The study was approved by the local ethics committee (Fondazione Policlinico “A. Gemelli” IRCSS, Rome, Italy – Università Cattolica del Sacro Cuore, Rome, Italy). The study involved human participants after a written informed consent, with guarantee of confidentiality and the permission to report individual data.

The protocol for the research project conformed to the provisions of the Declaration of Helsinki (as revised in Fortaleza, Brazil, October 2013).

The research reported in the manuscript neither received partial nor complete funding from commercial sponsors.

None of the contributing authors had any conflict of interest, including specific financial interests or relationships and affiliations relevant to the subject matter or materials discussed in the manuscript.

Each person listed as an author or co-author for this submitted manuscript met all the recommendations formulated by the International Committee of Medical Journal Editors regarding criteria for authorship.

This research study involving human subjects was registered in the publicly accessible ICMJE accepted registry: https://eudract.ema.europa.eu/ with EudraCT number: 2017-002585-43 issued for our Sponsor’s protocol code number EMDA/06/2017 (retrospectively registered on 17 June 2017).

All data are available on request.

## Results

Three out of 26 patients had early systemic adverse events due to EMDA®-MMC treatment leading to discontinuation of therapy; all of these 3 patients had recurrent papillary tumor at the enrollment and they had persistent high-grade disease at the endoscopic control after the discontinuation of the treatment, therefore they underwent radical cystectomy. All patients were considered for primary and secondary endpoints.

The median follow-up was 36 months (SD 3,4).

The high grade disease free rate at the end of follow-up was 61.5%.

At the end of the induction course, 3 patients (11.5%) had a low-grade tumor recurrence and we considered this partial response as a success of the induction EMDA®-MMC course. These patients therefore underwent further monthly intravesical maintenance EMDA®-MMC course and all of them were high grade disease-free at 3 years follow-up.

After 3 months of follow up (45 days after the end of the induction cycle), 5 patients (19.2%) underwent early radical cystectomy for high grade recurrent disease (including the 3 patients with early allergic reaction due to EMDA®-MMC), and 2 patients (7.7%) for disease progression.

After 9 months of follow up (45 days after the end of the maintenance cycle) 1 patient (3.8% of the total, 6.2% of the 16 patients treated with maintenance cycle) had HGNMIBC recurrence while 1 patient (3.8% of the total, 6.2% of the subgroup) had disease progression.

After 12 months of follow up 1 patient (3.8% of the total, 6.2% of the 16 patients treated with maintenance cycle) had disease progression; none of the patients experienced recurrent disease.

Subsequently, no other cases of recurrence or progression were reported until the end of follow-up.

After 3 years of follow-up, 10 patients (43.5%) underwent radical cystectomy, in 23.1% of cases (6 patients) for recurrent HGNMIBC (1 patient for TaG3, 3 patients for T1G3, 1 patient for Cis, 1 patient for T1G3 + Cis). Four patients (15.4% of cases) underwent radical cystectomy for progression to muscle-invasive disease, in 3 cases (13%) for pT2 disease (1 patient had initial T1G3, 1 patient had initial Cis, 1 patient had initial TaT1G3 + Cis) and in 1 case (3.8%) for pT4a (involvement of prostatic stroma at the level of prostatic urethra) (this patient had initial T1G3 + Cis) (Table 2).

**Table 2:** Indications to radical cystectomy after EMDA-MMC treatment

Recurrent HGNMIBC, n° of patients (%)	6 (23.1%)
TaG3	1 (3.8%)
T1G3	2 (7.7%)
Cis	1 (3.8%)
Ta/T1G3 + Cis	2 (7.7%)
Evidence of MIBC, n° of patients (%)	4 (15.4%)
T2	3 (11.5%)
T4	1 (3.8%)

Stratifying patients on TNM classification (TaG3, T1G3, Cis and Ta/T1G3 + Cis), high grade disease-free rates were 75, 78.6, 75 and 50%, respectively, at first follow-up; 75, 71.4, 75 and 25%, respectively, at 9 months follow-up; 75, 71.4, 50 and 25%, respectively, at 12 follow-up and until the end of follow-up.

**Fig. 1:**
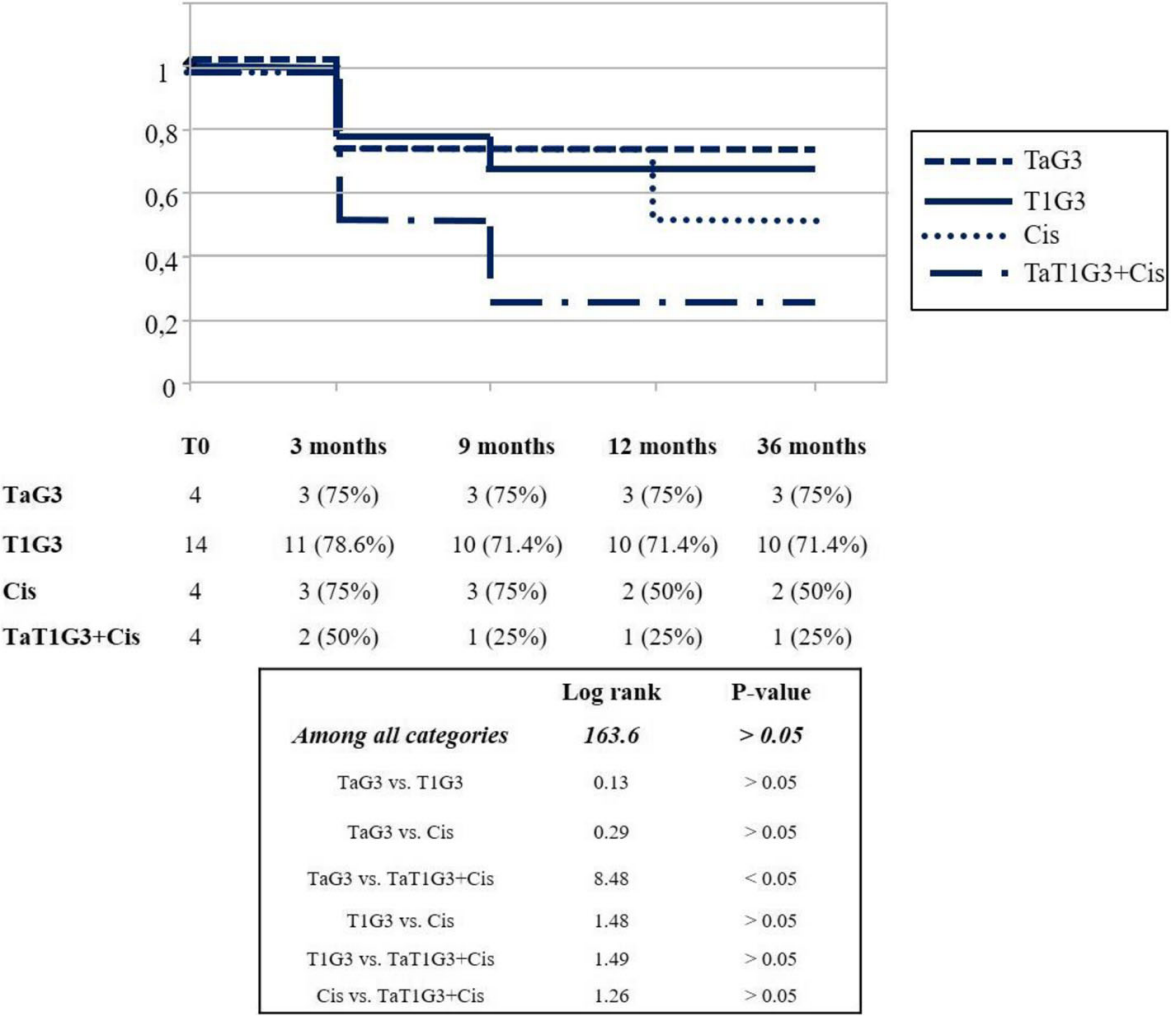
High grade disease-free survival Kaplan-Meiers curves based on stratification according stage/grade

Even if Kaplan-Meier analysis reported statistically significant differences in the relative survivals curves of all categorized patients (log rank = 163.6, *p* < 0.05, according to Chi-square Distribution Table), by pairwise comparisons to generate *p*-values between each possible comparison group, we reported statistically significant difference in relative survival curves only between patients with TaG3 vs TaT1G3 + Cis (Fig. 1).

**Fig. 2:**
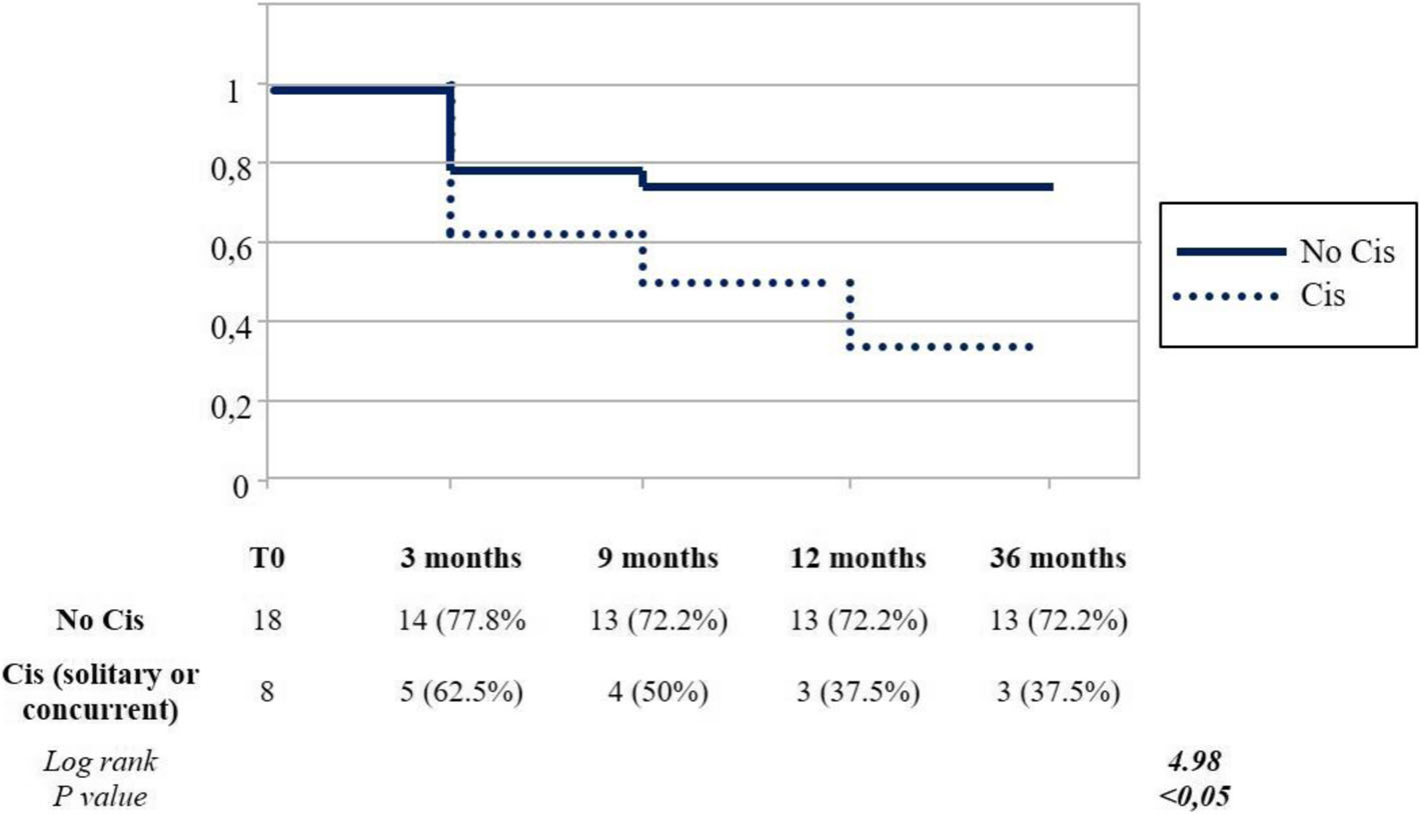
High grade disease-free survival Kaplan-Meiers curves based on stratification according the presence of Cis

By analyzing patients with Cis (solitary or concurrent in case of Ta/T1G3 + Cis) vs patients without Cis (TaG3 and T1G3 patients) we reported worse response rates in the first group (log rank = 4.98, p < 0.05) (Fig. 2).

Regarding toxicity (Table 3), 3 patients (11.5%) had a severe adverse systemic event of hypersensitivity to MMC with a hand-foot reaction; in these cases the treatment was discontinued and patients underwent allergy testing that confirmed the aetiopathogenesis of the adverse event.

**Table 3:** Adverse events

Systemic, n° of patients (%)	3 (11.5%)
Local, n° of patients (%)	6 (23.1%)
Pain	3 (11.5%)
Bladder spasm	3 (11.5%)
Dysuria	5 (15.4%)
Hematuria	1 (3.8%)
Difficult catheter insertion	1 (3.8%)
Frequency/urgency	3 (11.5%)
Nocturia	2 (7.7%)

Six patients (23.1%) had local side effects: Bladder spasms and suprapubic pain during the first instillation were treated and prevented before subsequent treatments with analgesic and antispastic drugs, while post-treatment dysuria and frequency regressed in 24 h after appropriate symptomatic therapy.

## Discussion

The prevention of recurrence and progression are the main aims of the therapy of HGNMIBC. BCG represents the gold standard but up to 50% of patients experience recurrence and up to 30% a disease progression (BCG failure). If the tumor persists after at least one cycle of BCG, EAU guidelines suggest early radical cystectomy [1].

Brausi et al. evaluated the electromotive administration on patients with multifocal G1-G2 Ta-T1 urothelial bladder cancer: they reported no differences in terms of complete response after 8 weeks of treatment compared to treatments with EMDA®-MMC and passive MMC (40% vs 41.6%, respectively), but patients treated with EMDA®-MMC showed lower recurrence rates (60% vs 33%, respectively) and longer disease-free intervals (14.5 vs 10.5 months, respectively), with low additional side effects [20].

However, in a prospective study on 108 patients with BCG-naïve multifocal high-risk NMIBC treated with three different regimens (EMDA®-MMC, MMC and BCG), the complete response rates at 3 and 6 months after EMDA®-MMC were significantly higher than the MMC (58 and 53% for EMDA®-MMC, 31 and 28% for MMC, respectively), but almost equivalent compared to BCG (64 and 56% respectively). Moreover data related to toxicity showed a higher rate of side effects with EMDA®-MMC than passive MMC, but significantly lower than BCG [21].

The “BCG-failure” represents a real challenge in clinical practice. The literature data showed the possible efficacy of the device-assisted intravesical therapies in case of HG-NMIBC unresponsive to BCG [37] but few studies and with a short follow-up are focused on the use of adjuvant EMDA®-MMC in case of BCG-failure HG-NMIBC (Table 4).

**Table 4:** Previous studies

Reference	N° of Pts	Tumor Stage	Response rate
Brausi M et al., Urology 1998 [20]	13	Ta-T1 Marker lesion	40% (at 6 months)
Di Stasi MS et al., J Urol 2003 [21]	108	BCG-naïve multifocal Cis	52.8% (at 6 months)
Sockett L et al., BJUInt 2008 [36]	13	BCG-refractory HGNMIBC	31% (at 15 months)
Riedl CR et al., J Urol 1998 [39]	16	Ta-T1	56% (at 14.1 months)
Our experience	23	BCG-refractory HGNMIBC	61.5% (at 36 months)

Sockett et al. reported complete response rates of 31% in absence of disease progression in a group of 13 patients with HG-NMIBC unresponsive to BCG, at 15 months of follow-up [36]. In our experience the high grade disease free rates were 73.1% at the end of the induction cycle and 61.5% after 3 years of follow up, with a progression rate of 15.4%. The EMDA®-MMC treatment after BCG-failure showed encouraging results. In terms of response rate (but not of the disease progression) these results appeared close to the previous study by Sockett et al. [36]: there are no more data available in this specific population, confirming the potential role of EMDA®-MMC as a useful tool in terms of a second-line bladder sparing treatment in BCG-failure. In our experience more than 60% of patients showed a benefit from EMDA®-MMC treatment preserving their bladder. Moreover, with the stratification according to the pathological stage, patients with Cis, primary or concurrent, showed worse oncological outcomes even with EMDA®-MMC.

In the previous cited study by Di Stasi et al. [21], authors evaluated a subgroup of 13 patients who underwent two intravesical BCG courses prior to EMDA®-MMC treatment. After 12 months of follow-up, only 3 (23.1%) patients obtained cancer remission (with no data on bladder preservation) compared to 16 (61.5%) patients in our study who obtained cancer-free status and bladder preservation after 3 years follow-up. These data might further corroborate our results, both because the number of patients examined in our study was double (with greater statistical power), and because in our study the pathological stage stratification provided more information on effectiveness of the EMDA®-MMC in these selected population of patients.

Regarding toxicity, in our experience, the rate of severe adverse events (allergic reaction with hypersensitivity to MMC) (11.5%) was acceptable and not as high as we expected and it was not so different from data reported in literature; in the study of Di Stasi et al. [21] the rate of allergic reaction was 8.3% on 36 patients treated with EMDA®-MMC. Regarding local side effects related to EMDA®-MMC, we reported lower rates than those reported by Di Stasi et al., for example in terms of hematuria (22.2% vs 3.8% in our data), urinary frequency (19.4% vs 11.5%) and dysuria/drug induced cystitis (36.1% vs 15.4%). Furthermore, the rate of discontinuation of treatment schedule in our study appeared slightly higher (11.5% vs 8.3%) to the rate reported by the same authors.

EMDA®-MMC is not approved by the EMA nor by the FDA, yet.

We think that our experience might be relevant and helpful for clinicians and even guidelines panel about the use and implementation of EMDA®-MMC technology in selected patients with HGNMIBC.

We followed the IBCG recommendations [38] on appropriate clinical trial designs in HGNMIBC based on current literature and expert consensus, in order to provide clinically relevant data, choosing specific eligibility criteria and performing baseline evaluations.

Moreover we specified the type of BCG-failure in order to make comparisons across trials feasible. Our results in terms of complete response rate (for carcinoma in situ) and recurrence-free rate (for papillary tumours) were non inferior compared to recommended clinically meaningful data: at least 50% at 6 months, 30% at 12 months, and 25% at 18 months.

As recommended, we used recurrence-free survival as the primary end point and toxicity as secondary end point; we evaluated progression rate as primary end point for its high clinical relevance in this selected population.

In the nonrandomized setting, the sample size of our study was calculated based on the disease-free survival rate at a fixed point in time using a one-stage Fleming design.

On the basis of these values, we calculated the required number of patients and the minimum number of patients who should be disease free for the EMDA®-MMC to be probably worthy of further studies. Therefore, on the basis of number of patients in the subgroups (with and without Cis), the rates of complete response in our study appeared in line and were not inferior (50% in our study as in IBCG recommendation for Cis at 6 months, and 72.2% in our study vs 30% for IBCG recommendation for papillary disease at 1 year).

Limitations of this study are represented by the small number of patients selected in a single center with the risk of a low statistical power and the lack of a control arm. However, the results obtained as a pilot study and the presence in literature of only one other study focused on the use of EMDA®-MMC as second line therapy in BCG-failure patients and with long follow-up [36] encourage us to continue the experience.

## Conclusions

The EMDA®-MMC might be considered safe and effective in the field of conservative treatment of high risk NMIBC unresponsive to BCG. The overall results seemed to be promising and could provide critical data for the further development of this therapeutic strategy.

Multicenter studies with larger number of patients and longer follow-up might justify a wider use of EMDA®-MMC, a technique with acceptable impact in term of costs and side effects than other already well established device-assisted treatments.

The HGNMIBC non responsive to BCG is an indication for early radical cystectomy; hence “bladder sparing” treatment with EMDA®-MMC could play a potential role in selected patients. We therefore propose to continue the enrollment and to prolong follow-up in order to give greater significance to our experience.

## Abbreviations

BCG: Bacillus of Calmette-Guerin
CR: complete response
EMDA: Electro-Motive Drug Administration
HGNMIBC: high grade non muscle invasive bladder cancer
MIBC: muscle invasive bladder cancer
MMC: Mitomycin C
NP: neoplastic progression
PR: partial response
SD: standard deviation
TURBT: trans-urethral resection of bladder tumour
